# Acknowledgement to Reviewers of *Biology* in 2018

**DOI:** 10.3390/biology8010007

**Published:** 2019-01-24

**Authors:** Biology Editorial Office

**Affiliations:** MDPI, St. Alban-Anlage 66, 4052 Basel, Switzerland

Rigorous peer-review is the corner-stone of high-quality academic publishing. The editorial team greatly appreciates the reviewers who contributed their knowledge and expertise to the journal’s editorial process over the past 12 months. In 2018, a total of 48 papers were published in the journal, with a median time to first decision of 21 days and a median time to publication of 52 days. The editors would like to express their sincere gratitude to the following reviewers for their cooperation and dedication in 2018:
Abou-Shanab, RedaKriete, AndresAdam, PaulKrogh, NicolaiAliverti, AlessandroKrupovic, MartAmir, MohammedKudo, FumitakaAmirkhani, MasoumeKumar, PrasunAzuma, MizukiKunkle, BrianBackert, SteffenKyriacou, CharalambosBarca, AmilcareLafont, FrankBerman, Sarah B.Leung, IvanhoeBertrand, MartineLevin, MichaelBideshi, DennisLi, YanBishop, KarenLopez-Ferber, MiguelBohutskyi, PavloMaceiras, RocíoBorger, PieterMancianti, FrancescaBovolenta, PaolaMarie-Orleach, LucasBruno, LauraMartins, IsabelCastanera, RaúlMazzulli, JoeChen, ChaoMcDaneld, Tara G.Chen, Chun-LiangMcDonald, GrantChen, Yu-ChihMcGuinness, DagmaraCholeris, ElenaMehrshahi, PayamClayton, AndrewMiletta, MariaCleaves, Henderson JamesMiller, Robert H.Coe, Christopher L.Mirey, GladysColwell, ChrisMitchell, KevinDarzynkiewicz, ZbigniewMontell, CraigDasgupta, SantanuMoonens, KristofDe Bernard, MarinaMouget, Jean-LucDe Ninno, AntonellaMuller, MarcDegola, FrancescaMüller, PeterDeryusheva, SvetlanaNeely, Melody N.Dissel, StephaneOphir, RonDolga, AmaliaPatel, DevenDong, YiboPavan, StefanoDramsi, ShaynoorPohl, EhmkeElias, Damian OctavioPolidoros, AlexiosEngel, Paul C.Ponnambalam, SreenivasanFachet, MelaniePopham, Holly J.Fainsod, AbrahamRachek, LyudmilaFan, Guo-ChangRamos-Suárez, Juan LuisFoulkes, NickReddy, HemachandraFowler-Finn, KaseyRizos, DimitriosFrankenberg, StephenRoy, KasturiFujii, RitsukoRubio, VicenteFujimori, KoRudrawar, SantoshFunk, RichardRuffing, Anne M.Furuya, ToshikiRuiz, MarioGallaher, Sean DavidSato, ShinGarcía-González, MercedesSelvaratnam, ThineshGerber, MarietteShuker, DavidGoubault, MarlèneShultz, Jeffrey W.Griffith, M. PatrickShumskaya, MariaGuerrero, Miguel G.Siniscalco, DarioGurrapu, SreeharshaStessman, Holly A.Hardwick, J. MarieSu, YanyanHarwood, Steven M.Sun, KaiHausman, Gary. J.Szatmari, ErzsebetHemanathan, KumarTirosh, ItayHeubel, KatjaToft, SørenHill, Peggy S. M.Trivellin, GiampaoloHiroi, NoboruVarela, SusanaHoffmann, KlausVavitsas, KonstantinosJones, AliceVervaet, BenjaminKaldis, PhilippViebahn, ChristophKhozin-Goldberg, InnaVitali, JacquelineKiecker, ClemensWelgamage Don, AakashKim, Jin-KyungWinuthayanon, WipaweeKim, Sung-HoonXiu, ShuangningKlassen, ViktorYoung, RosannaKleinegris, DorindeYurkov, Andrey M.Ko, ChemyongZarka, AlizaKojima, ShihokoZimba, PaulKoller, MartinZuccolo, Andrea

